# An asymmetric inhibition model of hemispheric differences in emotional processing

**DOI:** 10.3389/fpsyg.2014.00489

**Published:** 2014-05-23

**Authors:** Gina M. Grimshaw, David Carmel

**Affiliations:** ^1^School of Psychology, Victoria University of WellingtonWellington, New Zealand; ^2^Psychology Department, University of EdinburghEdinburgh, UK

**Keywords:** frontal asymmetry, hemispheric asymmetry, emotion, executive control, attention, EEG, prefrontal cortex, inhibition

## Abstract

Two relatively independent lines of research have addressed the role of the prefrontal cortex in emotional processing. The first examines hemispheric asymmetries in frontal function; the second focuses on prefrontal interactions between cognition and emotion. We briefly review each perspective and highlight inconsistencies between them. We go on to describe an alternative model that integrates approaches by focusing on hemispheric asymmetry in inhibitory executive control processes. The asymmetric inhibition model proposes that right-lateralized executive control inhibits processing of positive or approach-related distractors, and left-lateralized control inhibits negative or withdrawal-related distractors. These complementary processes allow us to maintain and achieve current goals in the face of emotional distraction. We conclude with a research agenda that uses the model to generate novel experiments that will advance our understanding of both hemispheric asymmetries and cognition-emotion interactions.

## HEMISPHERIC ASYMMETRIES IN EMOTIONAL PROCESSING

Prefrontal cortex (PFC) plays a critical role in emotion, but we are just starting to understand how complex interactions within the PFC give rise to emotional experience. One productive line of research examines hemispheric differences in emotional processing, focusing primarily on electroencephalography (EEG) studies of individual differences in frontal asymmetry as indexed by alpha oscillations. Alpha power has long been assumed to be negatively correlated with cortical activity (Pfurtscheller et al., 1996; Klimesch, 1999; Coan and Allen, 2004); this has led to the convention of describing left and right frontal *activity* as inverse of left and right frontal alpha power. Commonly, frontal asymmetry is measured as a trait (usually in the resting state) and is associated with a number of clinical, personality, and emotional factors, sometimes collectively called affective style (Davidson, 1992, 1998; Wheeler et al., 1993). Relatively low left (compared to right) frontal activity is associated with withdrawal-related traits including depression and anxiety (Thibodeau et al., 2006), shy temperament (Fox et al., 1995), dispositional negative affect (Tomarken and Davidson, 1994), and poor regulation of negative emotions (Jackson et al., 2003). In contrast, relatively low right (compared to left) frontal activity is associated with approach-related traits including dispositional positive affect (Tomarken and Davidson, 1994), trait anger (Harmon-Jones and Allen, 1998), sensation-seeking (Santesso et al., 2008), and high reward sensitivity (Harmon-Jones and Allen, 1997; Pizzagalli et al., 2005).

Frontal asymmetry does not, in general, correlate with current mood state, but with vulnerability or propensity to experience a particular state. For example, relatively low left frontal activity is observed in remitted depression (Henriques and Davidson, 1990; Gotlib et al., 1998), in the infants of depressed mothers (Field and Diego, 2008), and in those with genetic or familial risk of the disorder (Bismark et al., 2010; Feng et al., 2012). It also predicts future depression in healthy individuals (Nusslock et al., 2011). The predictive strength of frontal asymmetry led Davidson (1992, 1998) to propose that it reflects a diathesis – a characteristic way of processing emotional information which, when combined with sufficient stress, leads to disorder.

Two models have tried to capture the fundamental difference between hemispheres. The valence hypothesis (Tomarken et al., 1992; Heller, 1993; Heller et al., 1998; Berntson et al., 2011) grounds emotional asymmetry in affect, and associates left frontal cortex with positive emotion and right frontal cortex with negative emotion. The alternative motivational direction hypothesis (Harmon-Jones and Allen, 1997; Sutton and Davidson, 1997; Harmon-Jones, 2003) grounds emotional asymmetry in action, and associates left frontal areas with motivation to approach, and right frontal areas with motivation to withdraw. These models have sparked decades of research and produced a catalog of traits, behaviors, and biomarkers that are correlated with different patterns of asymmetry (for reviews, see Coan and Allen, 2004; Harmon-Jones et al., 2010; Rutherford and Lindell, 2011).

We see two limitations with both models. The first is that they are premised on the assumption that there is a fundamental frontal asymmetry that should explain all findings. Given the diverse functions of prefrontal cortex and the complex nature of emotional processing, that assumption seems unlikely to hold (see also Miller et al., 2013). It is useful here to consider a potential analogy with language asymmetries, which exist at the levels of phonology, syntax, semantics, and prosody; each subserved by separate neural systems. Although there are overarching principles of hemispheric organization for language, the asymmetries themselves are at least partially dissociable. A second limitation is that both models are largely descriptive. Neither specifies the mechanisms that are lateralized, or explains how they give rise to either emotion or motivation. We again see precedent established in language research, where progress was made when researchers focused on hemispheric asymmetries in the component processes of language instead of global language function. In this perspective, we draw on emerging understanding of cognition-emotion interactions within prefrontal cortex to propose the asymmetric inhibition model, which focuses on asymmetries in executive control mechanisms that allow us to control our emotions so that we can meet current goals.

## COGNITION-EMOTION INTERACTIONS IN PREFRONTAL CORTEX

The past decade has seen much progress in describing the complex interplay among brain networks that subserve emotion (for reviews, see Lindquist et al., 2012; Ochsner et al., 2012; Pessoa, 2013). To summarize, the generation of an emotional response begins with subcortical structures (including amygdala and ventral striatum) that are sensitive to the presence of behaviourally relevant stimuli. These structures modulate attention to the stimulus (Padmala et al., 2010; Pourtois et al., 2013), and activate a sequence of physiological responses that prepare us to approach or withdraw (Lang and Bradley, 2010). Orbito-frontal cortex (OFC) receives input from subcortical structures and sensory cortex, and computes emotional appraisal, tagging the stimulus as either punishment or reward in the context of one’s current needs (Rolls, 2004; Kringelbach, 2005). Anterior insula (AI) integrates this information with afferent projections from the body, giving rise to emotional awareness or feeling (Craig, 2009; Gu et al., 2013). Ventro-medial PFC (vmPFC) is closely associated with emotional experience and evaluation of emotional relevance for the self (Ochsner et al., 2004).

Lateral regions of PFC, together with anterior cingulate cortex (ACC), have traditionally been linked to cognitive functions, but contemporary models include these as core aspects of emotional processing (Gray et al., 2002; Ochsner and Gross, 2005; Pessoa, 2008, 2013; Dolcos et al., 2011). Ventro-lateral regions (vlPFC) support response selection and inhibition, and are part of the bottom–up ventral attention network that orients attention to behaviourally-relevant (including emotional) stimuli (Corbetta and Shulman, 2002; Viviani, 2013). Dorso-lateral regions (dlPFC) are involved in processes that provide top–down cognitive control, including working memory and the executive functions of updating, shifting, and inhibition (Kane and Engle, 2002; Miyake and Friedman, 2012). They are also part of the top–down dorsal attention network that directs attention in goal-relevant ways (Corbetta and Shulman, 2002; Vossel et al., 2014). Both dlPFC and vlPFC are active during forms of emotion regulation that are cognitively mediated, including cognitive reappraisal (Ochsner et al., 2012), and attentional control over emotional distraction (Bishop et al., 2004; Hester and Garavan, 2009). Sometimes dorsal and ventral regions act reciprocally, reflecting a trade-off between the ventral emotion system and the dorsal executive system (Dolcos and McCarthy, 2006; Dolcos et al., 2011; Iordan et al., 2013). However, the regions sometimes act in concert, as during cognitive reappraisal (Ochsner et al., 2012) and attentional control (e.g., Bishop et al., 2004). The exact pattern of interaction may depend on task demands and the ways in which emotional distractors compete with goal-relevant information for executive control (Pessoa, 2013). Generally, increased activation in dlPFC is associated with decreased activation in amygdala and ventral striatum (Beauregard et al., 2001; Davidson, 2002; Bishop et al., 2004; Ochsner et al., 2012), although these regions are not directly connected (Ray and Zald, 2012). Rather, dlPFC likely achieves its regulatory effects either via connections to vlPFC (Wager et al., 2008), or indirectly through control of attentional and semantic processes (Banich, 2009; Banich et al., 2009) that alter how emotional stimuli are perceived and interpreted (Ochsner et al., 2012; Vossel et al., 2014).

Hemispheric asymmetry does not figure prominently in current theories of prefrontal function in emotion. One reason might be methodological; most data come from fMRI studies that are rarely designed to assess asymmetry. When asymmetries are reported, they are often incidental to the experimental design and based on findings of significant activation in one hemisphere but not the other. However, to determine if the hemispheres differ from each other it is necessary to directly compare activation in homologous regions (Jansen et al., 2006). Such analyzes are common in studies of language asymmetries (e.g., Jansen et al., 2006; Cai et al., 2013), but rare in studies of emotion. A second issue is that there are far more studies of negative than positive emotional processing, meaning that meta-analyzes are dominated by negative studies (e.g., Phan et al., 2002; Wager et al., 2003; Ochsner et al., 2012) and individual studies rarely include both positive and negative stimuli. Unless both valences are represented, it is impossible to determine whether any observed hemispheric differences are related to valence or to emotional processes more generally.

Even given these caveats, there is little compelling evidence for asymmetries related to the *generation* of emotional experience. Amygdala activity is asymmetric; however, the asymmetry is related to stimulus properties, with the left more active for verbal and the right for visual representations (Costafreda et al., 2008; McMenamin and Marsolek, 2013). OFC is organized along a lateral gradient, with rewards represented in medial areas and punishers in lateral areas (Kringelbach, 2005), but again with no reliable hemispheric asymmetries related to either valence or motivational direction. Studies in which emotions are induced show bilateral activation of medial PFC regardless of valence (Phan et al., 2002; Wager et al., 2003). Multivoxel pattern analysis (e.g., Kassam et al., 2013; Kragel and LaBar, 2014), shows that there are distinct patterns of activity associated with positive and negative emotional experience, but these are broadly and bilaterally distributed across ventro-medial and orbito-frontal regions. There is, however, some evidence for asymmetries in the cognitive control of emotion associated with lateral PFC (Wager et al., 2003; Ochsner et al., 2012). We return to this below.

## THE ASYMMETRIC INHIBITION MODEL

The absence of consistent asymmetries in fMRI studies stands in contrast to robust findings of emotion-related asymmetries in EEG studies. How can we reconcile these findings? We start with an important observation – that EEG asymmetries are seen in alpha power. The assumption underlying all EEG asymmetry research is that alpha is inversely correlated with cortical activity. Therefore, asymmetric alpha levels are taken to reflect greater cortical activity in the hemisphere with lower alpha (Coan and Allen, 2004). This assumption is overly simplistic and does not reflect current knowledge of either the differentiation of prefrontal networks or the functional role of alpha oscillations. Few studies of EEG asymmetry use source localisation procedures, but those that have done so localize alpha asymmetries to dlPFC (Pizzagalli et al., 2005; Koslov et al., 2011). More generally, studies that measure simultaneous EEG and resting state fMRI find alpha to be inversely correlated with activity in the dorsal fronto-parietal network that coordinates activity between dlPFC and posterior parietal cortex (Laufs et al., 2003; Mantini et al., 2007) and plays an important role in the top–down executive control of attention (Corbetta and Shulman, 2002), primarily through modulations of sensory processing (for review, see Vossel et al., 2014). Functionally, alpha oscillations play a key role in attentional control and gating of perceptual awareness (Hanslmayr et al., 2011; Mazaheri et al., 2013).

The strong association between alpha and the fronto-parietal network leads us to propose that EEG asymmetries reflect the integrity of executive control mechanisms that inhibit interference from irrelevant emotional distractors. Executive control holds goal-relevant information in working memory in order to prioritize attention to relevant (over irrelevant) information (Desimone and Duncan, 1995; Kane and Engle, 2002; Lavie, 2005). Emotional stimuli are strong competitors for processing resources – this is adaptive, because they have such high behavioral relevance. But sometimes success depends on our ability to ignore the emotional stimulus and get on with the task at hand. With the Asymmetric Inhibition Model, we propose that mechanisms in left dlPFC inhibit negative distractors, and those in right dlPFC inhibit positive distractors. As we detail below, the model both accounts for much existing data and yields specific, testable predictions about how manipulations of executive control should affect hemispheric asymmetry.

## EXISTING EVIDENCE FOR THE MODEL

Our goal here is not to systematically review all research on emotional asymmetry (see comprehensive reviews by Coan and Allen, 2004; Harmon-Jones et al., 2010; Rutherford and Lindell, 2011). Rather, we provide examples to demonstrate that many existing asymmetries can be interpreted in terms of executive control. In the clinical literature, for example, trait EEG asymmetries predict vulnerability to several emotional disorders that are also characterized by difficulties with executive control. Those that are associated with relatively low left frontal activity (such as depression and anxious arousal) entail difficulty in disengaging attention from negative information (Eysenck et al., 2007; Cisler and Koster, 2010; De Raedt and Koster, 2010; Gotlib and Joormann, 2010). Poor self-regulation and addiction, both associated with relatively low right frontal activity, entail difficulty in inhibiting positive distractions (Bechara, 2005; Garavan and Hester, 2007; Goldstein and Volkow, 2011).

In experimental contexts, the model predicts that EEG asymmetries should be correlated with ability to control emotional distractions. Although most EEG studies focus on personality traits or emotional responses, a few recent studies have tested relationships between trait asymmetry and attention. In all studies, emotional faces were used as cues, but the facial expressions themselves were task-irrelevant. In a spatial cueing task, people with low left frontal activity showed difficulty disengaging from angry (but not happy) faces (Miskovic and Schmidt, 2010). In our own lab (Grimshaw et al., under review) we found similar results using a dot-probe task, which can be used to indicate the capture of attention by an emotional stimulus. Participants with low left frontal activity had difficulty shifting attention away from angry (but not happy) faces, but those with high left frontal activity were unaffected by the faces. Pérez-Edgar et al. (2013) had participants perform the same dot-probe task after an emotional stressor. Those who responded to the stress by increasing left frontal activity showed no attentional biases in the dot-probe task, but those who failed to do so showed biases to angry (but not happy) faces. All these studies are consistent with the idea that left frontal activity, as measured in EEG, reflects of the ability to recruit executive control processes that inhibit negative distractions when they are contrary to current goals.

Neuroimaging studies provide some evidence consistent with the model, if we are mindful of the caveats identified in Section ”Cognition-Emotion Interactions in Prefrontal Cortex”. We focus on studies in which the emotional stimulus or dimension is task-irrelevent and must be ignored (e.g., emotional Stroop, irrelevant emotional flankers). These tasks consistently produce greater activation for emotional than neutral distractors in dlPFC, and often in vlPFC. Compton et al. (2003) found increased activation in left dlPFC during presentation of negative words in an emotional Stroop task. Failure to recruit left dlPFC in the face of negative distraction has been associated with depression (Engels et al., 2010; Herrington et al., 2010), anxiety (Bishop et al., 2004), trait negative affect (Crocker et al., 2012) and schizotypy (Mohanty et al., 2005). Positive stimuli (including erotica, foods, and addiction-related cues) can also tax executive control processes (Pourtois et al., 2013). Control over positive distractions is commonly associated with activity in right vlPFC (Beauregard et al., 2001; Hester and Garavan, 2009; Meyer et al., 2011) and sometimes in right dlPFC (Beauregard et al., 2001).

Across these EEG and neuroimaging studies, there is stronger support for left lateralization in the inhibition of negative stimuli than right lateralization in the inhibition of positive stimuli, even in studies that used both positive and negative stimuli (e.g., Compton et al., 2003; Pérez-Edgar et al., 2013). This is problematic for our model, because support depends critically on the hemisphere by valence interaction. One possible explanation for this imbalance is that most studies of emotional distraction have used emotional faces or words as stimuli. Although these stimuli can be matched on *subjective* ratings of arousal, negative words and faces typically produce more behavioral interference than positive stimuli (Pratto and John, 1991; Horstmann et al., 2006), suggesting that they are more taxing for executive control systems. A better test of the model would use positive and negative stimuli such as pictures of scenes, which have equivalent potential to attract and hold attention (e.g., Schimmack, 2005; Vogt et al., 2008). Consistent with this speculation, the studies that associate inhibition of positive distraction with right lateral PFC all use emotional pictures as stimuli.

As correlational methods, EEG and fMRI cannot establish causal relationships between neural activity and function. However, brain stimulation methods, including transcranial magnetic stimuluation (TMS) and transcranial direct current stimulation (tDCS) can directly alter neural function and so establish causality. In clinical research, activation of left dlPFC with both TMS and tDCS is effective in the treatment of depression (Kalu et al., 2012). Consistent with the asymmetric inhibition model, treatment appears not to alter mood directly, but to improve executive control so that patients are better able to control negative biases (Moser et al., 2002). Conversely, right-sided stimulation affects motivation to approach positive stimuli. For example, activation of right dlPFC leads to reductions in both craving (Boggio et al., 2008; Fregni et al., 2008) and risky decision-making (Fecteau et al., 2007).

## AN AGENDA FOR FUTURE RESEARCH

We are not the first to suggest that emotional asymmetries reflect inhibitory processes (see Terzian, 1964; Jackson et al., 2003; Davidson, 2004; Coan et al., 2006, for explicit statements about asymmetries in inhibitory or regulatory functions). We extend this tradition by specifying a neurologically and cognitively plausible mechanism through which hemispheric differences in emotional processing might emerge. The asymmetric inhibition model draws on our increasingly sophisticated understanding of prefrontal function. In doing so, it not only provides explanation of many existing findings, but also suggests new experimental approaches that will move our conceptualization of emotional asymmetry beyond its current descriptive level.

The model argues for a shift in focus from the study of emotion *per se* toward the study of executive processes that are subserved by lateral PFC and the dorsal fronto-parietal network. Experiments should draw on the rich literature in cognitive psychology that has identified ways to target specific components of executive control. A simple but useful paradigm involves use of irrelevant distractors (e.g., Forster and Lavie, 2008). The “goal” is an emotionally neutral task, such as finding a target letter in a display that is flanked by irrelevant distractor images, which can be either emotional or neutral. One can then manipulate the availability of executive control in order to assess its role in inhibition. For example, increasing working memory load decreases the availability of executive control and its ability to inhibit irrelevant distractors (Lavie et al., 2004; Hester and Garavan, 2005; Carmel et al., 2012). Conversely, motivational manipulations enhance relevance of the goal and increase ability to inhibit distractors (Pessoa, 2009; Hu et al., 2013). These paradigms can be used in combination with fMRI and EEG recordings to determine whether positive and negative distractions are controlled by dissociable mechanisms, and whether those are differentially lateralized.

Because of inherent limitations in EEG and fMRI approaches, stimulation studies using TMS and tDCS are important for establishing causal relationships between prefrontal function and emotional inhibition. Brain stimulation may be particularly useful in hemispheric asymmetry studies, because it provides access to higher order frontal processes that are not as amenable to experimental manipulations (such as lateralized perceptual input) that have been used to study asymmetries in other domains. The asymmetric inhibition model makes specific predictions about the effects of lateralized stimulation on inhibition. Activation of left dlPFC should improve ability to inhibit negative (but not positive) distractions; activation of right dlPFC should improve ability to inhibit positive (but not negative) distractions.

The asymmetric inhibition model differs from other accounts of emotional asymmetry in two ways. First, it does not associate an entire hemisphere with a specific emotional or motivational state; rather it focuses on one asymmetry in a single mechanism, allowing it to generate specific and testable predictions. Second, the model turns conventional wisdom on its head; associating left PFC with the inhibition of withdrawal (instead of the support of approach), and right PFC with the inhibition of approach (instead of the support of withdrawal). The model is therefore consistent with current work on cognition-emotion interactions that emphasizes the role of lateral PFC in inhibitory executive control. Although we have shown here the value of incorporating cognition-emotion interactions into models of hemispheric asymmetry, we also think that models of cognition-emotion interaction would benefit from more careful consideration of hemispheric differences. Integration of perspectives should yield richer understanding of emotional processes.

## Conflict of Interest Statement

The authors declare that the research was conducted in the absence of any commercial or financial relationships that could be construed as a potential conflict of interest.
